# Case report: Mitochondrial trifunctional protein deficiency caused by *HADHB* gene mutation (c.1175C>T) characterized by higher brain dysfunction followed by neuropathy, presented gadolinium enhancement on brain imaging in an adult patient

**DOI:** 10.3389/fneur.2023.1187822

**Published:** 2023-06-13

**Authors:** Ruoyi Ishikawa, Masahiro Nakamori, Megumi Takenaka, Shiro Aoki, Yu Yamazaki, Akihiro Hashiguchi, Hiroshi Takashima, Hirofumi Maruyama

**Affiliations:** ^1^Department of Clinical Neuroscience and Therapeutics, Hiroshima University Graduate School of Biomedical and Health Sciences, Hiroshima, Japan; ^2^Department of Neurology and Geriatrics, Kagoshima University Graduate School of Medical and Dental Sciences, Kagoshima, Japan

**Keywords:** MTP deficiency, peripheral neuropathy, higher brain dysfunction, brain calcification, cerebral gadolinium enhancement, CNS demyelination

## Abstract

Mitochondrial trifunctional protein (MTP) deficiency is an autosomal recessive disorder caused by impaired metabolism of long-chain fatty acids (LCFAs). Childhood and late-onset MTP deficiency is characterized by myopathy/rhabdomyolysis and peripheral neuropathy; however, the features are unclear. A 44-year-old woman was clinically diagnosed with Charcot-Marie-Tooth disease at 3 years of age due to gait disturbance. Her activity and voluntary speech gradually decreased in her 40s. Cognitive function was evaluated and brain imaging tests were performed. The Mini-Mental State Examination and frontal assessment battery scores were 25/30 and 10/18, respectively, suggesting higher brain dysfunction. Peripheral nerve conduction studies revealed axonal impairments. Brain computed tomography showed significant calcification. Magnetic resonance imaging revealed an increased gadolinium contrast-enhanced signal in the white matter, suggesting demyelination of the central nervous system (CNS) due to LCFAs. The diagnosis of MTP deficiency was confirmed through genetic examination. Administration of L-carnitine and a medium-chain fatty triglyceride diet was initiated, and the progression of higher brain dysfunction was retarded within 1 year. This patient's presentation was suggestive of CNS demyelination. The presence of brain calcification, higher brain dysfunction, or gadolinium enhancement in the white matter in patients with peripheral neuropathy may be suggestive of MTP deficiency.

## 1. Introduction

Mitochondrial trifunctional protein (MTP) deficiency is an autosomal recessive disorder decreasing the enzyme involved in long-chain fatty acids (LCFAs) oxidation (1). The trifunctional protein is an octamer composed of four alpha and four beta subunits. The alpha and beta subunits are encoded by *HADHA* and *HADHB*, respectively (2). MTP deficiency presents heterogeneous clinical phenotypes varying from early-onset life-threatening cardiomyopathy, hypoketotic hypoglycemia, and liver failure to a late-onset form with myopathy, recurrent rhabdomyolysis, and peripheral neuropathy (3). In some late-onset cases, the symptoms of peripheral neuropathy are similar to those of Charcot–Marie–Tooth disease (CMT) (4, 5). Additionally, some cases are complicated by hypoparathyroidism (6, 7).

Herein, we describe the case of a 44-year-old patient who had been clinically diagnosed with hereditary neuropathy when she was a child. Recently, she developed progressive higher brain dysfunction and MTP deficiency was subsequently diagnosed. Brain magnetic resonance imaging (MRI) suggested demyelination of the central nervous system (CNS). This report aimed to highlight the characteristics of adult MTP, which can be complicated by progressive higher brain dysfunction due to demyelination of the CNS, including calcification and gadolinium enhancement in the white matter. It is possible to diagnose MTP deficiency in a patient with hereditary neuropathy, such as CMT.

## 2. Case description

A 44-year-old female (height, 1.56 m; weight, 49.5 kg) had been clinically diagnosed with hereditary neuropathy at the age of 3 due to gait disturbance. Among her two brothers, her older brother was clinically diagnosed with hereditary neuropathy and atypical CMT. When she was 10 years old, Achilles tendon lengthening was performed. She was able to walk until the age of 15 years, after which she was confined to a wheelchair. After graduating high school, she could not find a job and lived with her parents. Her muscle weakness progressed gradually. Based on the information we have collected, she had no childhood viral infections that might have induced a metabolic derangement and worsened the clinical symptoms. From the age of 43, she started stuttering and gradually stopped her hobbies such as writing, handicrafts, and games. At the age of 44 years, her response slowed with decreased activity and voluntary speech. As these symptoms progressed, she was referred to our hospital for a thorough investigation.

Physical examination revealed a dropped head, scoliosis, pes cavus feet, and a hammer toe. Muscle forces were evaluated using manual muscle testing. The proximal muscles in the bilateral upper and lower limbs revealed a score of 4. In her lower limbs, both tibialis anterior and triceps surae obtained a score of 1 or 2. The hand grip strength was 6 kg/4 kg (right/left). In the distal lower limbs, her pain and deep sensations were decreased. Muscle atrophy was observed in the distal lower limbs. Tendon reflexes were absent in the biceps brachii, triceps brachii, brachioradialis, patellar tendon, and Achilles tendon. Babinski and Chaddock signs were positive (big toe extension) in both feet. Finger-to-nose test of both hands revealed dysmetria. Gaze-evoked nystagmus was observed. Sentence recitation was sometimes challenging, and vocabulary recall was extremely slow. The Mini-Mental State Examination (MMSE) score was 25/30, and the frontal assessment battery (FAB) score was 10/18. These findings suggested higher brain dysfunction.

**Figure 1:**
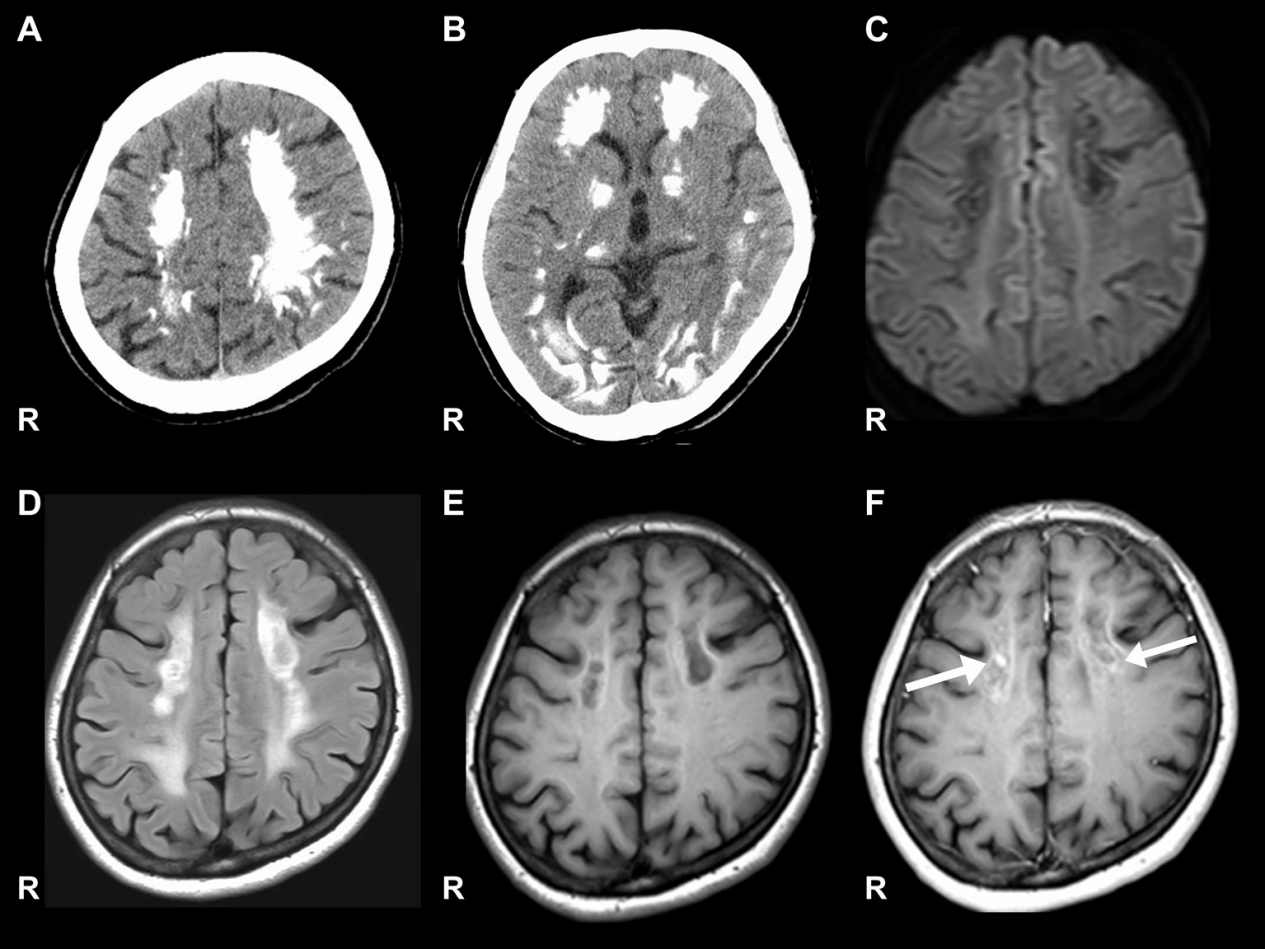
Brain CT and MRI. **(A, B)** Show significant calcification in the bilateral cortex, subcortex, and basal ganglia on computed tomography (CT). **(C)** Signal level is decreased in the white matter on diffusion-weighted images. **(D)** Signal level is increased in the white matter on fluid-attenuated inversion recovery imaging. **(E)** Signal level is decreased in the white matter of the frontal lobe on the T1-weighted image. **(F)** Gadolinium contrast-enhanced signal levels is increased in the white matter of the frontal lobe on the T1-weighted image.

**Figure 2:**
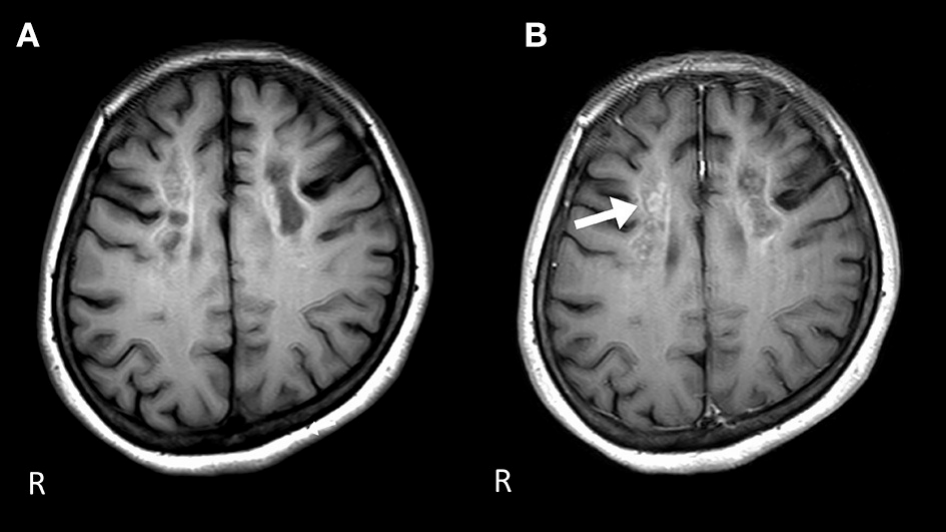
Brain MRI 1 year after the start of the therapy. **(A)** The signal level is decreased in the white matter of the frontal lobe on the T1-weighted image. **(B)** Gadolinium contrast-enhanced signal levels are still sustained compared to those 1 year ago in the white matter of the frontal lobe on the T1-weighted image.

**Figure 3:**
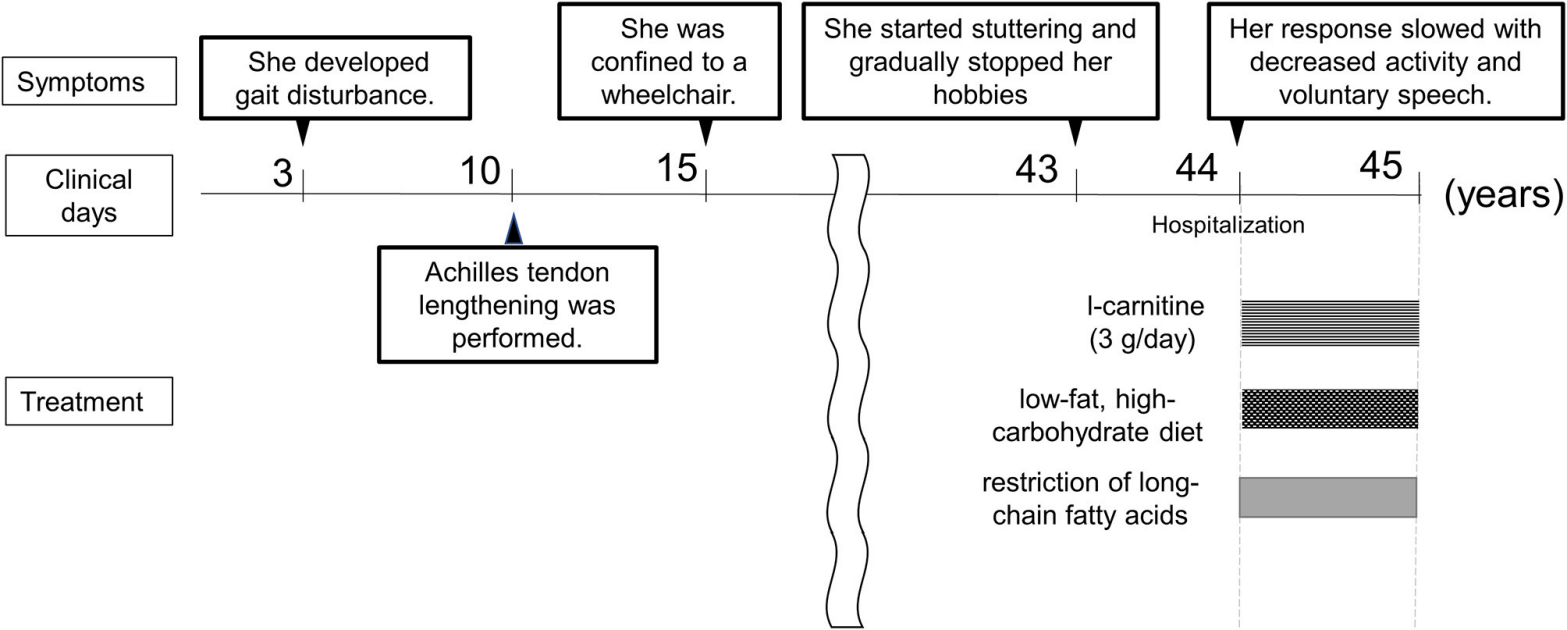
Clinical course. The time course of the patient is shown.

Nerve conduction studies (NCS) showed decreased compound muscle action potential (CMAP) and sensory nerve action potential (SNAP) amplitudes, sharing almost normal conduction velocity in peripheral nerves of the extremities, suggesting axonopathy. Ophthalmologic examination revealed no retinopathy. Brain computed tomography (CT) revealed significant calcification in the bilateral cortex, subcortex, basal ganglia, and cerebellar dentate nuclei. Skeletal muscle CT showed muscle atrophy and fatty degeneration of the lower limbs. Magnetic resonance imaging (MRI) showed increased fluid-attenuated inversion recovery signals in the white matter and increased gadolinium contrast-enhanced T1-weighted imaging (T1WI) signal in the white matter of the frontal lobe (Figure 1). Blood tests, including blood count, creatine kinase, calcium ion, phosphorus, parathyroid hormone, autoantibodies, and very long-chain fatty acid (VLCFA), were not remarkable. Her plasma total carnitine concentration was 28.9 μmol/L which was slightly low (normal range:45–91 μmol/L). Cerebrospinal fluid tests, including the oligoclonal band and lactate/pyruvate ratio, were also normal. Although the calcium, phosphorus, and parathyroid hormone levels were normal, significant brain calcification might suggest past hypoparathyroidism. “Calcification” and “peripheral neuropathy,” implied MTP deficiency. Acylcarnitine analysis revealed elevated long-chain acylcarnitines, such as C16-OH and C18:1-OH, which are characteristic of MTP deficiency. A genetic examination for MTP deficiency revealed a mutation, c.1175C>T [p.A392V], which was found in two alleles of the *HADHB* gene. Fluorescence *in situ* hybridization showed no abnormalities at PMP22, and comprehensive screening by next-generation sequencing of CMT showed no abnormalities. Based on these results, we diagnosed MTP deficiency (6). Subsequently, to prevent the progression of MTP deficiency, she was prescribed a low-fat, high-carbohydrate diet and restriction of long-chain fatty acids, including a medium-chain fatty triglyceride (MCT) diet. l-carnitine (3 g/day) was also administrated. (5). 1 year after treatment commencement, the MMSE score was 26/30, and FAB score was 11/18. Increased gadolinium contrast-enhanced T1WI signal was not spread; however, it was sustained (Figure 2). These findings suggested that the obvious progression of her higher brain dysfunction was not detected, but the demyelination was sustained. The clinical course of the patient's disease is described in Figure 3.

## 3. Discussion

In this case, the patient clinically would rather be regarded as a childhood-onset type (3). Childhood and late-onset MTP deficiency is usually characterized by myopathy/rhabdomyolysis and peripheral neuropathy. However, the most important and unique features of our patient were progressive higher brain dysfunction, brain calcification, and gadolinium enhancement in the white matter. Hence, elucidating the underlying mechanisms of these symptoms is important for gaining new insights into the pathophysiology of this disease.

It was reported that hypoparathyroidism improved with growth in two siblings with MTP deficiency (6). Similarly, our patient has the same mutation, c.1175C>T. In addition, there were two cases of brain calcification reported; however, there was no hypoparathyroidism during the diagnosis of MTP deficiency (4, 8). Our patient may have had subclinical hypoparathyroidism during childhood which improved with growth. Since parathyroid hormone and calcium ion levels were normal, it seemed that progressive calcification of the brain had already stopped in this patient. This is insufficient to explain progressive higher brain dysfunction due to the calcification of the brain. Therefore, we focused on the increased gadolinium contrast-enhanced T1WI signals in the white matter of the frontal lobe. Myelin in oligodendrocytes is composed mainly of fatty acids containing 16–26 carbons, which are abundant in the brain and crucial for brain function (9, 10). Before considering MTP deficiency, adrenoleukodystrophy (ALD) should be considered. ALD is a peroxisomal disorder due to the metabolic derangement of VLCFA which contains 22–28 carbons (11). VLCFA accumulation induces cerebral inflammatory demyelination due to progressive destabilization of myelin sheaths and causes higher brain dysfunction (11). ALD is characterized by an increased gadolinium contrast-enhanced signal in the white matter, similar to our patient (12). Compared to ALD, MTP deficiency impairs the metabolism of LCFAs which contain 14–21 carbons that are also crucial for brain function (13). In addition, among patients with MTP deficiency, accumulation of lipid droplets in the muscle has been reported (14). Theoretically, LCFAs would also accumulate in the white matter of patients with MTP deficiency, instead of VLCFA, since fatty acids that contain 16–21 carbons are also abundant in the brain.

Several pathomechanisms of MTP deficiency have been proposed such as the accumulation of toxic hydroxyl acylcarnitines, energy deficiency, oxidative stress, and disruption of membrane lipid composition (15, 16). It is possible that these factors will induce progressive destabilization of myelin sheaths and cerebral inflammatory demyelination. Therefore, progressive higher brain dysfunction might be due to demyelination of the CNS by LCFAs. This was a unique case since there have been no reports of MRI gadolinium enhancement in the white matter, which is thought to suggest inflammatory demyelination in patients with MTP deficiency.

Although there is a possibility that brain calcification could be a confounding factor in advanced disease with severe white matter degeneration, there are few reports that Fahr's disease, a representative disease with brain calcification, has gadolinium enhancement. Therefore, we suppose the gadolinium enhancement in this case is due to active demyelination. Further case series and pathological findings are needed to prove the demyelination of the white matter.

This patient was prescribed an MCT diet and l-carnitine (3 g/day). Fatty acid oxidation (FAO) disorder therapy, including MTP deficiency, is discussed in several previous reports (17–19). MTP deficiency requires a diet primarily avoiding LCFAs to prevent the accumulation of toxic hydroxyl acylcarnitines and oxidative stress. LCFAs are replaced with a specially produced medium-chain (C8) triglyceride formulation (MCT), which is efficiently chain-shortened by the medium-chain and short-chain FAO enzymes to produce energy (20). Carnitine supplementation can aid carnitine transport and subsequently improve MTP deficiency in theory; however, carnitine could also induce the accumulation of toxic intramitochondrial long-chain acylcarnitines, and supplementation has been associated with rhabdomyolysis (21). Since this patient did not present with rhabdomyolysis until now, we continued l-carnitine (3 g/day). If she develops rhabdomyolysis in the future, we would consider discontinuing carnitine.

This patient was clinically diagnosed with CMT during childhood. It is reported that the overall prevalence of neuropathy is 70% and the median age of onset of neuropathy is 4.7 years in MTP deficiency (22). In Japan, tandem mass spectrometry was introduced as a mass screening method for newborns in 2011, thus, the condition might have been overlooked in some adults. some cases of MTP deficiency may be hidden, which could be diagnosed as CMT in childhood. Even if the complications of myopathy/rhabdomyolysis are absent, the presence of significant brain calcification, progressive higher brain dysfunction, or white matter gadolinium enhancement in patients with peripheral neuropathy may lead to the diagnosis of MTP deficiency, which is treatable and should not be overlooked. Thus, our report will help in improving management strategies for such patients. As for the limitations of this report, it is noted that no examinations, including imaging, were performed from childhood until the patient presented here. It is desirable to comprehensively observe the temporal progression when diagnosing similar cases in the future. In particular, the contrast brain MRI findings, which are reported for the first time, are considered significant. The accumulation of further cases is desired.

## Data availability statement

The raw data supporting the conclusions of this article will be made available by the authors, without undue reservation.

## Ethics statement

Written informed consent was obtained from the individual(s) for the publication of any potentially identifiable images or data included in this article.

## Author contributions

RI and MN wrote the draft of the manuscript. RI, MN, MT, SA, YY, and HM examined this case. AH and HT provided comprehensive screening by next-generation sequencing of CMT. All authors contributed to the article and approved the submitted version.
